# Evaluation of a severity-based surgical strategy for refractory Mooren’s ulcer: A retrospective series with long-term follow-up

**DOI:** 10.1371/journal.pone.0359413

**Published:** 2026-09-30

**Authors:** Minghai Huang, Zhifeng Wu, Dongmei Wei, Jixiao Nong, Zhuoyuan Zhang, Thuthuy Hoang, Guina Yin

**Affiliations:** 1 Department of Ophthalmology, The Second Affiliated Hospital of Guangxi Medical University, Nanning, China; 2 Nanning Aier Eye Hospital, Nanning, China; University at Buffalo Jacobs School of Medicine and Biomedical Sciences: University at Buffalo School of Medicine and Biomedical Sciences, UNITED STATES OF AMERICA

## Abstract

**Objective:**

To evaluate the clinical outcomes of a Severity-Based Surgical Algorithm for treating refractory Mooren’s ulcer.

**Methods:**

We retrospectively reviewed consecutive patients with Mooren’s ulcer who underwent surgical intervention between February 2016 and January 2024 after failing medical therapy. A predefined severity-based surgical algorithm was applied: amniotic membrane transplantation (AMT) for ulcers involving <2/3 stromal thickness; lamellar keratoplasty (LK) for ulcers ≥2/3 thickness or perforations ≤4 mm; and penetrating keratoplasty (PK) for perforations >4 mm or technical infeasibility of LK. All patients received postoperative systemic and local immunosuppression. Primary outcomes included primary healing, changes in best-corrected visual acuity (BCVA), recurrence, and anatomical success.

**Results:**

The cohort comprised 24 patients (24 eyes) with a mean follow-up of 30 months (range 12–84). Seven eyes underwent AMT, 15 LK, and 2 PK. Primary healing rates were 100% for AMT and PK, and 93.3% for LK (overall 95.8%). Although BCVA improved or stabilized in 71.4% of AMT and 86.7% of LK eyes, functional acuity (BCVA ≥ 0.3) was achieved in 71.4% (5/7) of the AMT group compared to only 13.3% (2/15) of the LK group. Conversely, PK outcomes were poor (HM to NLP). Recurrence rates were 28.6% (2/7) for AMT, 64.3% (9/14) for LK, and 100% (2/2) for PK. Kaplan-Meier analysis showed a median recurrence-free survival of 36 months for the LK group. Crucially, final anatomical success was achieved in 85.7% of AMT, 93.3% of LK, and 50.0% of PK eyes, resulting in an overall globe preservation rate of 87.5% (21/24).

**Conclusion:**

A severity-graded surgical approach effectively preserves globe integrity in refractory Mooren’s ulcer. While AMT provides excellent outcomes for less-severe cases, cases requiring keratoplasty demonstrate more aggressive disease biology with higher recurrence risks. Therefore, vigilant long-term follow-up and sustained immunomodulation are mandatory for severe cases.

## Introduction

Mooren’s ulcer is a rare, painful, and idiopathic peripheral ulcerative keratitis characterized by progressive stromal melting and undermining of the corneal epithelium and stroma [1–3]. Although the precise etiology remains elucidated, converging evidence implicates an autoimmune basis. The clinical course is notoriously aggressive; without timely intervention, the disease can rapidly progress to descemetocele, frank perforation, and catastrophic loss of vision or globe integrity. Management of Mooren’s ulcer is strictly time-sensitive. Standard first-line therapy involves intensive topical corticosteroids, conjunctival resection, and often systemic immunosuppression. However, a significant subset of cases proves resistant to these measures. In this study, we defined “refractory Mooren’s ulcer” as disease that persists or progresses despite comprehensive conservative therapy (specifically, maximal tolerated medical therapy combined with adjunctive conjunctival resection. For these patients, surgical escalation is not merely an option but a globe-saving necessity.

While various surgical techniques—ranging from amniotic membrane transplantation (AMT) to lamellar keratoplasty (LK) and penetrating keratoplasty (PK)—have been described, the optimal surgical timing and choice of procedure remain controversial [4,5]. Current literature largely focuses on the outcomes of individual techniques in isolation, offering limited guidance on how to match specific surgical interventions to the severity of the ulceration. This lack of consensus often leads to heterogeneous, experience-based decision-making, potentially resulting in undertreatment of severe perforations or overtreatment of superficial melting.

To address this therapeutic conundrum, we implemented a standardized, severity-based surgical algorithm that stratifies interventions according to ulcer depth, perforation size, and extent. The purpose of this study is to evaluate the long-term clinical outcomes of this algorithmic approach in a consecutive series of refractory cases, specifically analyzing its efficacy in achieving anatomical stability, minimizing recurrence, and preserving visual function.

## Materials and methods

### Study design

This retrospective cohort study was conducted at Nanning Aier Eye Hospital, China. We analyzed surgical outcomes in patients with refractory Mooren’s ulcer who underwent severity-based surgical interventions between February 2016 and January 2024. The study was approved by the Medical Ethics Committee of Nanning Aier Eye Hospital (ID: NNAIER-2024-08) and adhered to the tenets of the Declaration of Helsinki. Written informed consent forms were obtained from all participants. The data used in this retrospective study were accessed for research purposes on 02 April 2024. All data were de-identified before analysis.Investigators did not have access to information that could identify individual participants at any time.

### Eligibility criteria

Medical records of all patients diagnosed with Mooren's ulcer at our institution from February 2016 to January 2024 were reviewed.

**Inclusion criteria:**

1. Clinical diagnosis of Mooren's ulcer, defined by a characteristic peripheral, crescentic corneal ulcer with overhanging edge, in the absence of scleritis or systemic autoimmune disease;
2. Failure to respond to comprehensive medical therapy and adjunctive conjunctival resection (see protocol below).

Our step-up conservative management protocol was tailored to disease severity. Topical 1% prednisolone acetate was initiated at a frequency of 4 times daily and escalated up to hourly as needed. If the response was inadequate after 1 week, adjunctive conjunctival resection was performed, followed by oral prednisolone (1 mg/kg/day) if clinical stabilization was not achieved within 3–7 days. Severe cases (e.g., bilateral involvement or extensive circumferential ulceration without impending perforation) received immediate concurrent topical and systemic corticosteroids. Oral cyclosporine A (5 mg/kg/day) was added as a steroid-sparing agent for refractory cases.

To ensure objective assessment, “refractory Mooren’s ulcer”—which was defined as the failure of comprehensive medical therapy and adjunctive conjunctival resection, and served as the absolute indication for reconstructive surgery (AMT, LK, or PK)—was strictly defined as:

(1) progressive ulceration and corneal thinning despite at least 3 weeks of the aforementioned maximum tolerated medical therapy and adjunctive conjunctival resection, or

(2) rapid corneal melting with impending or actual perforation at any time during active therapy.

3.Underwent surgical intervention with AMT, LK, or PK.

**Exclusion criteria:**

1. Clinical or laboratory evidence of alternative causes of peripheral ulcerative keratitis (e.g., rheumatoid arthritis, granulomatosis with polyangiitis, collagen vascular diseases);
2. Ocular infection or concomitant scleritis;
3. Incomplete clinical data or follow-up <12 months.

The diagnostic workup for all patients included: complete blood count, erythrocyte sedimentation rate, C-reactive protein, rheumatoid factor, anti-cyclic citrullinated peptide antibody, antinuclear antibody, anti-neutrophil cytoplasmic antibody, anti-Sjögren's syndrome-related antigen A/B antibodies, venereal disease research laboratory test, and chest radiography. Corneal smear and culture were also performed to exclude infectious keratitis. These tests were conducted to rule out systemic autoimmune diseases known to cause peripheral ulcerative keratitis, such as rheumatoid arthritis, systemic lupus erythematosus, and granulomatosis with polyangiitis. For patients who experienced recurrence, the systemic autoimmune workup was repeated at least once during follow-up to rule out the emergence of an initially occult systemic disease.

To ensure data independence and avoid inter-eye correlation bias, only one eye per patient was analyzed. For patients with bilateral presentation, we only included the eye that failed comprehensive conservative management and required surgical intervention (AMT, LK, or PK).

### Treatment protocol and surgical decision algorithm

#### Severity-based surgical algorithm and procedures.

The Severity-Based Surgical Algorithm is illustrated in Fig 1. Surgical modality was stratified by ulcer depth and perforation characteristics, assigning patients to amniotic membrane transplantation (AMT), lamellar keratoplasty (LK), or penetrating keratoplasty (PK). All procedures included meticulous debridement of necrotic tissue and excision of a 3-mm margin of perilimbal conjunctiva and Tenon’s capsule adjacent to the ulcer. A single experienced surgeon (M.H.) performed all operations under peribulbar or general anesthesia.

**1. Amniotic Membrane Transplantation (AMT)**

**Fig 1:**
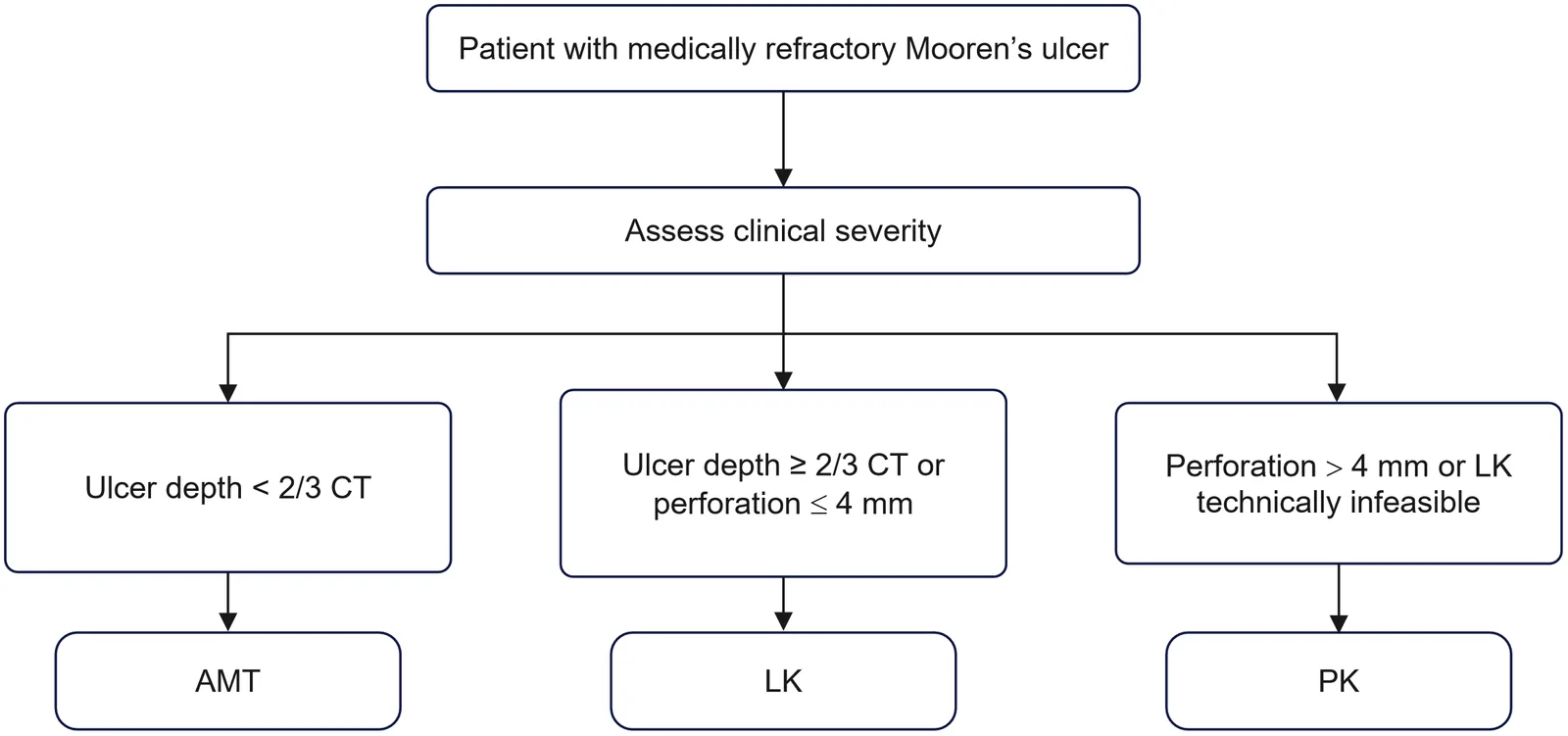
Severity-based surgical algorithm. Abbreviations: AMT, amniotic membrane transplantation; LK, lamellar keratoplasty; PK, penetrating keratoplasty.

Indication: Ulcer depth < 2∕3 of corneal thickness without perforation.

Technique: Under peribulbar anesthesia, the ulcer bed was gently debrided. A multilayered (6–10 layers) cryopreserved amniotic membrane was trimmed to fit the defect, with the basement-membrane side up to facilitate epithelial growth. The graft was secured with interrupted 10−0 nylon sutures, and a bandage contact lens was applied.

**2. Lamellar Keratoplasty (LK)**

Indications: Ulcer depth ≥ 2∕3 thickness, descemetocele, impending perforation, or perforation ≤4 mm.

2.1 For perforations 2–4 mm: a double-layer LK technique was used. This approach was modified from the technique described by Chen J et al. [2]. Briefly, a thin fresh endothelial lamellar patch (approximately 100 µm thick, 0.5 mm larger than the perforation diameter) was placed endothelial-side down to seal the perforation, secured with interrupted 10−0 nylon sutures. The anterior chamber was then re-formed by iris repositioning and injection of balanced salt solution or air. Second, an oversized outer lamellar corneoscleral button, trimmed according to the shape of the lesion (0.25 mm larger than the recipient bed, 400–500 µm thick), was sutured over the inner patch and onto the recipient bed with interrupted 10−0 nylon.
2.2 For ulcers without or with minimal perforation (<2 mm): conventional single-layer anterior LK was performed without an inner graft.
**3. Penetrating Keratoplasty (PK)**

Indications: PK was reserved for perforations > 4 mm or when LK was technically infeasible.

Technique: Under general anesthesia, the full-thickness recipient cornea was excised by manual dissection extending 0.25 mm beyond the ulcer margin. A full-thickness donor corneal button, oversized by 0.25 mm relative to the excised recipient bed and including a 1–2 mm lamellar scleral rim, was fashioned to match the contour of the excised cornea and secured to the host bed with interrupted 10−0 nylon sutures. The anterior chamber was reconstituted with balanced salt solution.

### Postoperative management

All patients received a standardized postoperative regimen. Topical therapy included 0.5% levofloxacin four times daily and tobramycin–dexamethasone ointment nightly for 1 month. In addition, 1% prednisolone acetate was administered four times daily for the first month, then tapered gradually over 6–12 months. Systemic immunosuppression consisted of oral prednisolone (1 mg/kg/day, tapered gradually over 1–2 month) combined with cyclosporine A (5 mg/kg/day in two divided doses, tapered gradually over 3–6 month). Specifically for patients who underwent LK or PK, topical therapy was transitioned to 0.1% fluorometholone once daily after 1 year for long-term maintenance, individualized according to recurrence risk.

Recurrences were first managed by reapplying and intensifying the initial therapeutic approach—up to hourly topical 1% prednisolone acetate, conjunctival resection, and reinitiation of systemic immunosuppression as indicated. Persistent or progressive disease despite this regimen (i.e., refractory per our predefined criteria) was subsequently re-evaluated for surgery using the same severity-based surgical algorithm.

### Follow-up and outcome measures

Patients were followed for 12–84 months. Serial evaluations included visual acuity testing, intraocular pressure measurement (non-contact or iCare tonometry), slit-lamp biomicroscopy, and fluorescein staining. Ancillary tests—including anterior segment optical coherence tomography, in vivo confocal microscopy, and microbiological cultures—were performed as clinically indicated. Specifically, in vivo confocal microscopy was employed to rapidly exclude opportunistic infectious etiologies (such as fungal hyphae and Acanthamoeba cysts) in cases of suspected corneal superinfection, and to evaluate subbasal/stromal dendritic cell density and morphology to provide objective microstructural guidance during the initiation and subsequent tapering of immunosuppressiv therapy. Outcomes assessed comprised:

**Primary outcomes:**

Primary Healing: Defined as complete corneal epithelialization (negative fluorescein staining) combined with the cessation of stromal thinning.

Recurrence: Defined as the re-emergence of marginal ulceration, infiltration, or progressive stromal thinning in the host cornea, graft or at the graft-host junction after initial primary healing had been achieved. This was strictly differentiated from immunological graft rejection based on clinical signs.

Anatomic Success: Defined as the preservation of globe integrity without the need for evisceration or enucleation, and the absence of phthisis bulbi at the final follow-up, regardless of graft clarity.

**Secondary outcomes:**

Secondary outcomes included best-corrected visual acuity (BCVA), and postoperative complications such as complicated cataract, secondary glaucoma, and allograft rejection.

### Statistical analysis

Descriptive statistics summarized demographic and clinical data (mean ± SD or median [IQR] for continuous variables; counts and percentages for categorical variables). Kaplan–Meier survival analysis estimated the cumulative recurrence-free probability following LK; only the first recurrence was analyzed as the endpoint. Statistical analyses were performed with IBM SPSS Statistics version 22 (IBM Corp., Armonk, NY, USA).

## Results

### Patient demographics and clinical characteristics

Between February 2016 and January 2024, a total of 52 patients (66 eyes) with Mooren's ulcer were assessed for eligibility. Among them, 26 patients (26 eyes) with refractory disease underwent surgical reconstruction (AMT, LK, or PK). After excluding two patients due to insufficient follow-up (<12 months), the final cohort comprised 24 patients (24 eyes) (Fig 2). To ensure data independence and avoid inter-eye correlation bias, only the eye requiring surgical intervention after failing conservative management was included for the 8 patients with bilateral disease; the contralateral eyes in these cases were successfully managed with conservative therapy alone. For the analyzed cohort, the median follow-up was 30.0 months (IQR, 23.5–48.0 months; range, 12–84 months).

**Fig 2:**
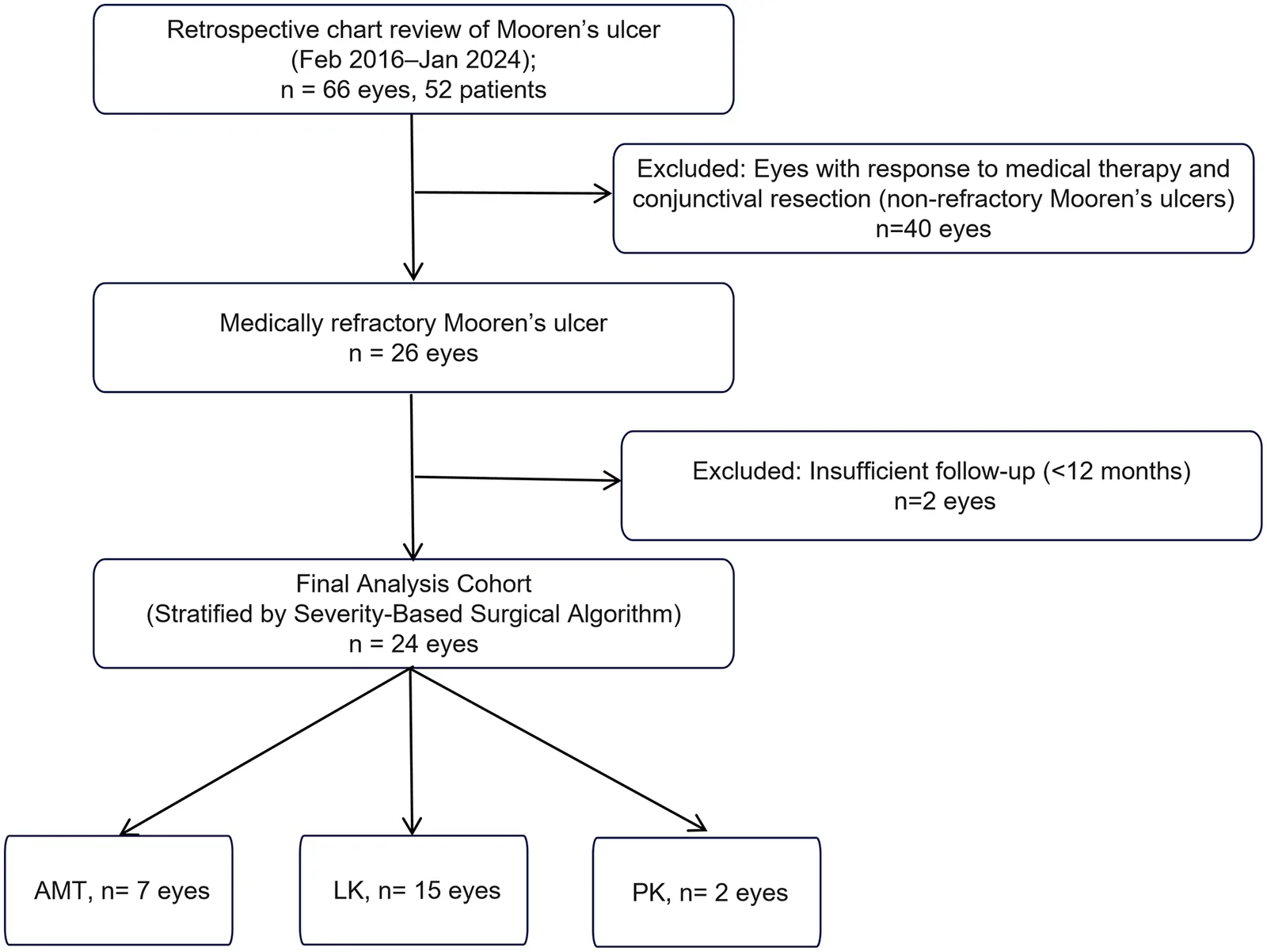
Study flow diagram illustrating participant selection and treatment allocation based on the severity-based surgical algorithm. Abbreviations: AMT, amniotic membrane transplantation; LK, lamellar keratoplasty; PK, penetrating keratoplasty.

The baseline demographic and clinical characteristics of the cohort are detailed in Table 1, and individual patient-level data are provided in S1 Table. Key overall indicators included a long median symptom duration prior to surgery (7.5 months), extensive peripheral involvement (≥6 clock hours in 66.7%), and a high rate of corneal perforation (45.8%). Preoperative best corrected visual acuity was correspondingly poor, with a median of 0.10 (logMAR 1.0; IQR, HM–0.45). Over half of the eyes (14/24, 58.3%) had a BCVA of 0.1 or worse, including 9 eyes with hand motion (HM) and 2 with finger counting (FC). Baseline characteristics differed logically across the surgical tiers, reflecting an individualized treatment strategy based on disease severity. Patients undergoing AMT generally presented with less severe, non-perforated ulcers and better preoperative vision. In contrast, those requiring lamellar keratoplasty (LK) and especially penetrating keratoplasty (PK) had more extensive circumferential involvement, deeper ulcers, and higher rates of perforation.

**Table 1:** Baseline demographics and preoperative severity by surgical tier.

Characteristic	AMT (n = 7)	LK (n = 15)	PK (n = 2)	Total (n = 24)
Age, years — median (IQR)	51 (48–60)	43 (34–55)	33, 60^a^	49 (37.5–57.5)
Male sex — n/N (%)	4/7 (57.1%)	8/15 (53.3%)	2/2 (100%)	14/24 (58.3%)
Bilateral disease — n/N (%)	1/7 (14.3%)	7/15 (46.7%)	0/2 (0%)	8/24 (33.3%)
Symptom duration, months — median (IQR)	3 (3–5)	12 (7–15)	4, 8^a^	7.5(4.0–12.5)
Preop BCVA (decimal) — Median (IQR)	0.5 (0.4–0.8)	HM (0.1–HM)^b^	HM, FC^a^	0.1 (0.45–HM)^b^
Preop BCVA (logMAR) — Median (IQR)	0.30 (0.10–0.40)	2.30 (1.00–2.30)^b^	2.30, 2.00^a,b^	1.00 (0.35–2.30)^b^
Clock hours involved — median (IQR)	4 (3–5)	7 (6–11)	9, 10^a^	6 (5.0–10.5)
Extensive involvement ≥6 h — n/N (%)	1/7 (14.3%)	13/15 (86.7%)	2/2 (100%)	16/24 (66.7%)
Central corneal involvement — n/N (%)	0/7 (0%)	9/15 (60.0%)	2/2 (100%)	11/24 (45.8%)
Ulcer depth category — n/N (%)				
— <2/3 CT	7/7 (100%)	0/15 (0%)	0/2 (0%)	7/24 (29.2%)
— ≥2/3 CT, non‑perforated (includes Descemetocele)	0/7 (0%)	6/15 (40.0%)	0/2 (0%)	6/24 (25%)
— Perforated	0/7 (0%)	9/15 (60.0%)	2/2 (100%)	11/24 (45.8%)
Perforation size, mm — median (range)^c^	—	2 (1–4)	4.5 (4–5)	3 (1–5)
Follow‑up, months — median (IQR)	25 (24–36)	36 (23–60)	12, 84^a^	30 (23.5–48.0)

Abbreviations: AMT, amniotic membrane transplantation; LK, lamellar keratoplasty; PK, penetrating keratoplasty; BCVA, best corrected visual acuity (decimal); CT, corneal thickness; HM, hand motion; FC, finger counting.

a, Individual values presented due to small sample size.

b, For statistical analysis, visual acuity was converted to logMAR; HM and FC were assigned logMAR values of 2.30 and 2.00, respectively. Medians and IQRs were computed on the logMAR scale and back-converted to decimal where shown for clinical interpretability.

c, Statistic applies only to the subset with corneal perforation (n = 11); not applicable (—) for the AMT group.

Note: Of the 24 patients, 8 had bilateral disease. To ensure data independence and avoid inter-eye correlation bias, only the surgically reconstructed eye (AMT, LK, or PK) was analyzed for each patient; contralateral eyes were successfully controlled with conservative management alone. Symptom duration refers to the interval from the onset of ocular symptoms (as reported by the patient) to the date of surgical intervention at our institution.

### Outcomes of surgical interventions

Surgical outcomes for each group are summarized below; individual patient-level data are provided in S1 Table.

### Amniotic membrane transplantation (AMT)

AMT was performed in 7 of 24 eyes (29.2%) for <2/3 CT, non-perforated ulcers, with a median follow-up of 25 months (IQR, 24–36 months) (Table 1).

This technique achieved universal initial healing and a high final anatomic success rate (85.7%); the integrated membrane provided robust tectonic support despite remaining opaque (Fig 3A,a), and a final BCVA of ≥0.3 was maintained in most eyes (71.4%). The primary adverse events were two ulcer recurrences (28.6%)—one controlled medically, the other requiring subsequent lamellar keratoplasty—and one case of postoperative cataract (Table 2).

**Fig 3:**
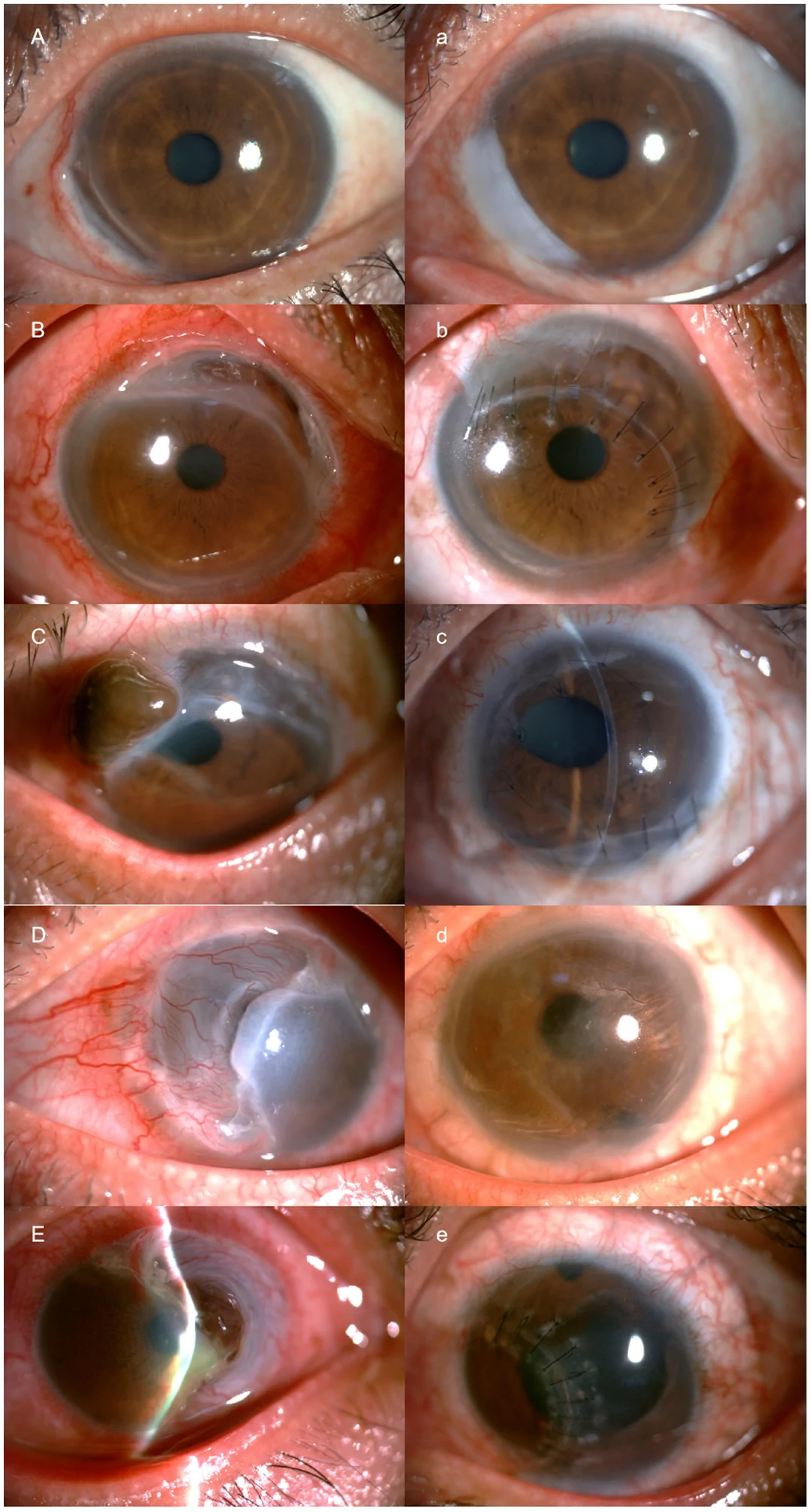
Representative preoperative (left) and postoperative (right) slit-lamp photographs illustrating surgical interventions selected according to the severity-based surgical algorithm for refractory Mooren’s ulcer. (A, a) AMT: The transplanted opaque amniotic membrane integrated with the recipient cornea, restoring structural integrity. (B, b) Partial LK: C-shaped LK for deep corneal ulcer presenting as descemetoceles, demonstrating graft transparency and restored corneal structural integrity. (C, c) Double-layer LK for a 4 mm corneal perforation with iris prolapse: A large D-shaped outer lamellar graft overlying a small, thin inner patch graft secured at the nasal perforation site, showing postoperative graft transparency and structural integrity. (D, d) Total LK for extensive peripheral corneal ulceration extending over 9 clock hours with central involvement, showing postoperative interface haze and vascularization while maintaining structural integrity. (E, e) Large C-shaped PK for extensive peripheral corneal ulceration spanning 9 clock hours with a 5 mm paracentral perforation, demonstrating mild postoperative graft edema while preserving corneal structural integrity and the recipient's nasal cornea. Abbreviations: AMT, amniotic membrane transplantation; LK, lamellar keratoplasty; PK, penetrating keratoplasty. Reprinted under a CC BY license, with permission from Nanning Aier Eye Hospital, original copyright 2016–2024.

**Table 2:** Outcomes by surgical tier in refractory Mooren’s ulcer.

Outcome	AMT (n=7)	LK (n=15)	PK (n=2)	Total (n=24)
Healing — n/N (%)	7/7 (100%)	14/15 (93.3%)	2/2 (100%)	23/24 (95.8%)
BCVA improved or stable — n/N (%)	5/7 (71.4%)	13/15 (86%)	0/2 (0%)	18/24 (75%)
Final BCVA ≧ 0.3 — n/N (%)	5/7 (71.4%)	2/15 (13.3%)	0/2 (0%)	7/24(29.2%)
Recurrence — n/N (%)	2/7 (28.6%)	9/14 (64.3%)*	2/2 (100%)	13/23(56.5%)*
Anatomic success — n/N (%)	6/7 (85.7%)	14/15 (93.3%)	1/2 (50%)	21/24 (87.5%)
Cataract — n/N (%)	1/7 (14.3%)	7/15 (46.7%)	2/2 (100%)	10/24 (41.7%)
Glaucoma — n/N (%)	0/7 (0%)	4/15 (26.7%)	0/2 (0%)	4/24(16.7%)
Graft rejection — n/N (%)	0/7 (0%)	0/15 (0%)	2/2 (100%)	2/24 (8.3%)

* One eye with failed initial healing after LK was excluded from the recurrence analysis. Abbreviations: AMT, amniotic membrane transplantation; LK, lamellar keratoplasty; PK, penetrating keratoplasty; BCVA, best corrected visual acuity (decimal).

### Lamellar keratoplasty (LK)

Lamellar keratoplasty was performed in 15 of 24 eyes (62.5%) for conditions including ulcer depth ≥ 2/3 of corneal thickness, descemetocele, or perforation. Patients were followed for a median of 36 months (IQR, 23–60 months) (Table 1). The surgical technique (partial, total, or double-layer graft) was selected based on ulcer severity—ulcer depth, perforation size and disease extent (Fig 3B,b–D,d).

Initial corneal healing was observed in 14 of 15 eyes. Final anatomic success rate was in 93.3% of eyes. Functional outcomes, however, were limited; only 13.3% of eyes achieved a final best corrected visual acuity (BCVA) of ≥0.3. Significant graft opacity developed in 73.3% of cases. Ulcer recurrence occurred in 64.3% of cases, with a median recurrence-free survival of 36 months by Kaplan-Meier analysis (Fig 4). Regarding the management of these recurrences, most were controlled with medical therapy. Two cases with milder ulceration were treated with amniotic membrane transplantation (AMT), while one eye progressed to perforation after the patient declined further intervention. Observed other complications were transient interface fluid, partial anterior synechiae (3 eyes, 20%), secondary glaucoma, and progressive cataract (Table 2).

**Fig 4:**
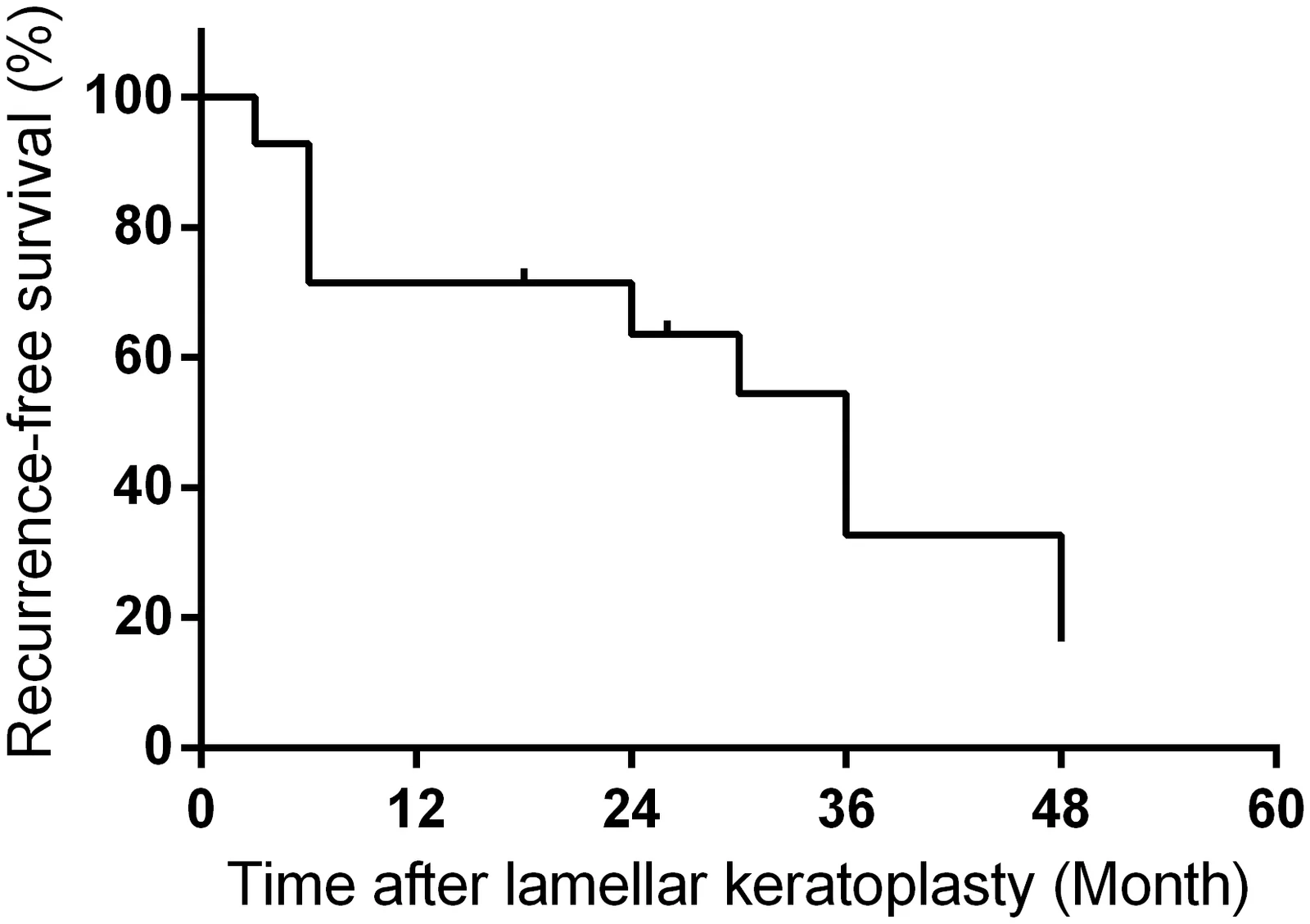
Kaplan-Meier analysis of recurrence-free survival after LK. Cumulative probabilities were 71.4%, 63.5%, 32.7%, and 16.3% at 12, 24, 36, and 48 months, respectively. The median recurrence-free survival time was 36 months (95% CI, 25–47 months). LK, lamellar keratoplasty.

**Penetrating Keratoplasty (PK)**:

PK was performed in two eyes (8.3%) for large perforations > 4 mm or when LK was technically infeasible, with follow-up periods of 12 and 84 months (Table 1; Fig 3E,e).

Both eyes initially healed, with a best recorded pre-rejection visual acuity of 0.1. However, both grafts ultimately failed. One eye developed rejection at 4 months and recurrence at 7 months postoperatively, progressing to perforation and phthisis bulbi (no light perception). The other experienced rejection at 6 months and recurrence at 24 months, culminating in graft failure with a final BCVA of hand motion. The anatomical success rate was 50% (1/2), while the rejection and recurrence rate was 100% (2/2) (Table 2).

## Discussion

Mooren’s ulcer remains therapeutically challenging because of its aggressive course and the absence of standardized treatment pathways. Although a stepladder immunosuppression strategy controls active disease in most patients [6–8], a clinically meaningful subset presents with refractory disease—often with impending or frank perforation—necessitating surgery to preserve globe integrity. Our study addresses this gap by validating a severity-stratified surgical algorithm. We demonstrated that matching the surgical modality to ulcer depth yielded high rates of globe preservation (87.5% overall). However, consistent with the chronic nature of the disease, durable prevention of recurrence remains the major unmet need. In our cohort, while initial healing was robust, recurrence occurred in 57.1% of eyes, with significantly higher rates in the keratoplasty groups (PK 100%, LK 64.3%) compared to AMT (28.6%). This divergence likely reflects, at least in part, the more severe baseline disease and longer follow-up duration in patients requiring keratoplasty. Cases so severe as to necessitate LK or PK require particularly vigilant long-term follow-up and sustained immunomodulation.

For non-perforated ulcers involving <2/3 corneal thickness, Amniotic Membrane Transplantation (AMT) served as the effective frontline intervention. Our AMT cohort achieved a final anatomical success rate of 85.7%, outcomes that are consistent with the membrane’s well-documented anti-inflammatory, pro-epithelialization, and low-immunogenic properties. These findings align with prior reports validating AMT in inflammation-mediated corneal ulceration [9,10].

Crucially, for a relapsing disease, functional preservation is as vital as tectonic success. By these metrics, AMT outperformed Lamellar Keratoplasty (LK): recurrence was lower (28.6% vs 64.3%) and visual outcomes were superior (BCVA ≥0.3 in 71.4% vs 13.3%), a finding consistent with comparative studies [11]. While one AMT recurrence ultimately required LK for tectonic stability, AMT also functioned effectively as salvage therapy for post-LK recurrences. Nevertheless, its efficacy is bounded by severity; consistent with reports of poor outcomes in highly aggressive disease [12], our protocol appropriately excluded extensive perforations from the AMT arm. Thus, current evidence supports AMT as the preferred initial intervention for less-severe refractory disease.

Despite being the most frequently performed procedure for Mooren's ulcer, LK outcomes remain highly variable. Our 64.3% recurrence rate was notably higher than previous reports: Chen et al. [2] achieved 25.6% recurrence using adjunctive cyclosporine, while Liu et al. [13] reported only 9.7% with confocal microscopy-guided immunosuppression. However, our results align more closely with recent studies involving severe cohorts, such as Yang et al. [14] (44.2%) and Ou et al. [15] (37.3%). Several factors explain this variability. First, and most importantly, this disparity reflects confounding by indication: our LK cohort exhibited exceptional disease severity, with 46.7% having bilateral “malignant” involvement [2,14], 60% presenting with perforation,and 86.7% had circumferential involvement ≥ 6 clock hours. Second, surgical and postoperative variables, including technique, medication regimens, and patient compliance, critically influence outcomes [13,15,16]. Third, our extended follow-up likely captured late recurrences missed in shorter studies. Within our severity-based strategy, LK was reserved for these advanced cases to provide immediate tectonic support—a critical objective where it demonstrated 93.3% anatomical success.

The double-layer LK technique offers a distinct advantage in perforated Mooren's ulcer by sealing the perforation with a thin inner lamellar patch while preserving the host endothelium and Descemet's membrane at non-perforated regions, thereby reducing antigenic load and exposure to a vascularized recipient bed, and lowering the risk of immunologic rejection compared with conventional large-diameter PK. In our series, this technique successfully managed perforations up to 4 mm, consistent with previous reports by Chen et al. [2] and Liu et al. [13]. Penetrating keratoplasty (PK) was therefore reserved strictly for large perforations where double-layer LK was technically unfeasible.

The poor outcomes in our PK group (100% failure due to recurrence/rejection) are unsurprising given the inflamed, vascularized recipient beds, increased graft size, and eccentric placement which collectively create high-rejection-risk conditions [17]. These results reinforce current practice favoring LK over PK, reserving full-thickness grafting only as a measure of last resort.

Kaplan-Meier analysis revealed a progressive decline in recurrence-free survival within the LK cohort, dropping from 71.4% at 12 months to 16.3% at 48 months. This temporal pattern underscores the chronic, relapsing autoimmune pathology of Mooren's ulcer, demonstrating that surgical intervention alone is insufficient to secure long-term remission. Crucially, recurrences frequently coincided with the tapering or discontinuation of immunosuppression, highlighting the necessity of sustained pharmacotherapy. For “severe-tier” patients, particularly those undergoing keratoplasty, maintenance immunosuppression must be prolonged to preserve disease quiescence, balancing efficacy against the potential for ocular and systemic toxicities through individualized risk-benefit optimization.

Although more potent immunomodulatory agents—including cyclophosphamide, methotrexate, mycophenolate mofetil, or biologics—might have enhanced long-term control, their implementation was constrained by the lack of a multidisciplinary rheumatology service and localized formulary restrictions. These limitations restricted our capacity to safely administer and monitor high-risk systemic therapies, occasionally impacting disease control in refractory cases. Ultimately, these challenges underscore the critical need for integrated, multidisciplinary care in managing complex, sight-threatening autoimmune ocular diseases.

### Study limitations

This study has several limitations inherent to its design as a retrospective, single-center case series. First, the relatively small sample size may limit the statistical power for subgroup comparisons; however, given the extreme rarity of Mooren's ulcer, obtaining a larger cohort of refractory cases at a single institution is challenging. Second, treatment allocation was based on our severity-based surgical algorithm rather than randomization, reflecting the intended clinical decision-making process in which patients with more severe disease underwent more extensive procedures. Direct comparisons of efficacy between surgical modalities should therefore be interpreted with caution, as outcomes are inherently influenced by baseline disease severity. Third, the limited sample size and number of recurrence events precluded meaningful multivariable Cox regression analysis without risking severe model overfitting. Our findings should therefore be interpreted as real-world descriptive outcomes of a severity-based surgical algorithm. These constraints underscore the need for future multi-center, prospective validation. Nevertheless, our findings provide vital real-world evidence supporting severity-based surgical decision-making, demonstrating that a structured, graded approach can effectively preserve globe integrity even in this challenging pathology.

## Conclusions

In conclusion, our severity-based surgical algorithm demonstrates that individualized treatment strategies can optimize outcomes. AMT represents the optimal intervention for mild-to-moderate disease, offering dual utility as primary and salvage therapy. LK effectively preserves globe integrity in advanced disease but carries a high recurrence risk, particularly in severe bilateral cases. PK should be reserved for cases where LK is technically unfeasible. Success mandates a paradigm shift: the necessity for keratoplasty should be viewed as a “red flag” for aggressive disease, necessitating sustained, individualized local and systemic immunosuppression to maintain long-term disease quiescence, thereby securing graft survival and preventing recurrence.

## Supporting information

S1 Table Individual Patient-Level Clinical Data of 24 Cases of Refractory Mooren’s Ulcer Treated with Severity-Based Surgical Algorithm. AMT, amniotic membrane transplantation; LK, lamellar keratoplasty; PK, penetrating keratoplasty; BCVA, best-corrected visual acuity (decimal); HM, hand motion; NLP, no light perception; FC, finger counting; CT, corneal thickness; Perf., perforation; OD, right eye; OS, left eye; B/L, bilateral; U/L, unilateral. Note: Of the 24 patients, 8 had bilateral disease. To ensure data independence and avoid inter-eye correlation bias, only the surgically reconstructed eye (AMT, LK, or PK) was analyzed for each patient; contralateral eyes were successfully controlled with conservative management alone. Symptom duration refers to the interval from the onset of ocular symptoms (as reported by the patient) to the date of surgical intervention at our institution. BCVA is presented in decimal notation. For statistical analysis, HM and FC were assigned logMAR values of 2.30 and 2.00, respectively. Em dash (—) in the “Time to First Recurrence” column indicates that no recurrence event occurred during follow-up. “NA” (not applicable) for Case 2 indicates that the eye was excluded from recurrence analysis due to failed initial healing after lamellar keratoplasty.
(XLSX)
